# OA26 A case of Weber-Christian disease: a diagnostic conundrum

**DOI:** 10.1093/rap/rkac066.026

**Published:** 2022-09-28

**Authors:** Diarmuid McLaughlin, Madeleine Rooney

**Affiliations:** Department of Paediatric Rheumatology, Musgrave Park Hospital, Belfast, Northern Ireland, United Kingdom; Department of Paediatric Rheumatology, Musgrave Park Hospital, Belfast, Northern Ireland, United Kingdom

## Abstract

**Introduction/Background:**

Weber-Christian Disease (WCD) is a rare idiopathic disorder characterised by panniculitis of adipose tissue that can present with fever, rash, myalgia, arthritis and can have multi-system involvement. It presents as a challenge to diagnose with patients often seen by other specialities prior to rheumatology. The management includes immunosuppression with multi-speciality input. There is debate about whether WCD is a distinct disease entity or is part of a wider disease spectrum of other disorders. This case highlights the diagnostic conundrum of WCD, its differential diagnosis and subsequent management. It also raises the need to recognise the potential multi-system involvement of WCD.

**Description/Method:**

A 3-year-old girl presented with weight loss, rash affecting the lower limbs and joint pains associated with morning stiffness. Past history included speech and language delay, pulmonary stenosis and fibroepithelial hyperplasia of the hard palate. Examination revealed an ejection systolic murmur, hepatosplenomegaly, muscle wasting of left buttock, facial redness, restriction of wrist movements with swelling and right knee swelling. Areas of lipo-hypertrophy were seen overlying both legs, lower back and arm regions.

Initial investigations included ESR 30 (mm/hr), CRP 17 (mg/L) with a normal FBC, iron profile, U&E, LFT, rheumatoid factor, CK, immunoglobulins and LDH. Virology and bacteriology screening panels were negative. Autoantibody screen (anti-ds DNA, ENA, ANA, mitochondrial, smooth muscle) was also negative. CXR, XR wrists, ultrasound of abdomen and right knee were normal.

Differential diagnosis included panniculitis, malignancy and auto-immune disorders including scleroderma. A biopsy was taken from the right calf, finding evidence of lymphocytic lobular panniculitis. A diagnosis of Weber Christian disease was subsequently made by the paediatric rheumatology team. The patient was commenced on chloroquine, prednisolone and omeprazole.

A genetics referral was completed due to concerns with aphasia and presence of a broad face. Microarray revealed a deletion of chromosome 10 (q21.2 – q22.2).

Over the next decade, the panniculitis settled on a combination of low dose prednisolone and chloroquine. However, a gradual deterioration in renal function was observed. Renal biopsy found evidence of focal segmental glomerulosclerosis eventually requiring haemodialysis and a subsequent successful renal transplant. Other issues included alopecia (treated with topical steroids), arthritis (managed with corticosteroid injections with limited success) and a large abdominal lipoma (resected by plastic surgery). The patient is now 19 years old with much resolution of the panniculitis and is managed on low dose prednisolone, hydroxychloroquine and omeprazole with mycophenolate, tacrolimus and amlodipine given for renal transplant management.

**Discussion/Results:**

Once other disorders were excluded following diagnostic work-up and biopsy, a diagnosis of panniculitis was made. WCD was the proposed working diagnosis given the clinical features and examination findings. The patient was subsequently successfully managed on immunosuppression including prednisolone, chloroquine which was later replaced by hydroxychloroquine. By monitoring urine dipstick at clinic appointments, microscopic proteinuria and haematuria was detected signifying renal involvement later resulting in a renal transplant.

Unusual features of this case include the young age of the patient at diagnosis (3 years old), the severity of renal disease involvement, the rarity of focal segmental glomerulosclerosis at the age of 8 years old and the lack of systemic features (fever) in this presentation of WCD. The identification of a chromosomal deletion is particularly relevant as it raises the question of the significance of this deletion on disease presentation, causation and severity. Expert clinical genetic opinion has suggested that the deletion is highly likely to be significant in relation to the speech delay of our patient and as a possible contributing cause of the patient’s panniculitis. Owing to the rarity of this condition and the genetic findings in our patient, the management was based upon a small evidence base of literature and multi-speciality involvement. Review of the literature on WCD discusses whether WCD is a distinct disease entity or not and argues it should be considered as part of a wider disease spectrum of other related disorders.

**Key learning points/Conclusion:**

This case highlighted the importance of multi-speciality involvement particularly in complex and unusual disorders. Often, these disorders do not follow a textbook description and require an open mind to consider the wider differential diagnosis and subsequent management. Early explanation with the patient’s parents about the rarity of the disorder and the complexities of its management was essential. Identifying the most suitable pharmacological agent to manage panniculitis in WCD is based upon a limited evidence base and in our case was achieved with prednisolone, chloroquine and later hydroxychloroquine. Further exploration on suitable treatments for WCD is important along with further discussion on the multi system involvement of this rare disorder.

